# Extracellular Vesicles (EVs) and Pancreatic Cancer: From the Role of EVs to the Interference with EV-Mediated Reciprocal Communication

**DOI:** 10.3390/biomedicines8080267

**Published:** 2020-08-03

**Authors:** Sokviseth Moeng, Seung Wan Son, Jong Sun Lee, Han Yeoung Lee, Tae Hee Kim, Soo Young Choi, Hyo Jeong Kuh, Jong Kook Park

**Affiliations:** 1 Department of Biomedical Science and Research Institute for Bioscience & Biotechnology, Hallym University, Chunchon 24252, Korea; sokvisethmoeng@yahoo.com (S.M.); miyanae@naver.com (S.W.S.); alex0827@naver.com (J.S.L.); gksdudsd@gmail.com (H.Y.L.); kimyou1009@naver.com (T.H.K.); sychoi@hallym.ac.kr (S.Y.C.); 2 Department of Medical Life Sciences, College of Medicine, The Catholic University of Korea, Seoul 06591, Korea; hkuh@catholic.ac.kr

**Keywords:** extracellular vesicles, exosomes, microvesicles, microenvironment, non-coding RNA, pancreatic cancer

## Abstract

Pancreatic cancer is malignant and the seventh leading cause of cancer-related deaths worldwide. However, chemotherapy and radiotherapy are—at most—moderately effective, indicating the need for new and different kinds of therapies to manage this disease. It has been proposed that the biologic properties of pancreatic cancer cells are finely tuned by the dynamic microenvironment, which includes extracellular matrix, cancer-associated cells, and diverse immune cells. Accumulating evidence has demonstrated that extracellular vesicles (EVs) play an essential role in communication between heterogeneous subpopulations of cells by transmitting multiplex biomolecules. EV-mediated cell–cell communication ultimately contributes to several aspects of pancreatic cancer, such as growth, angiogenesis, metastasis and therapeutic resistance. In this review, we discuss the role of extracellular vesicles and their cargo molecules in pancreatic cancer. We also present the feasibility of the inhibition of extracellular biosynthesis and their itinerary (release and uptake) for a new attractive therapeutic strategy against pancreatic cancer.

## 1. Introduction

Pancreatic cancer is an incurable and threatening malignancy that is the seventh major cause of cancer mortality worldwide in 2018 [1]. Patients with pancreatic cancer commonly present local or distant metastasis upon diagnosis—and the limited efficacy of anticancer therapies, such as chemotherapy and radiotherapy, often leads to the recurrence of cancer and its associated death up to the present [2]. Therefore, it is critical to identify and develop new treatment approaches to strive against this disease to resolve this serious matter.

The microenvironment in pancreatic cancer consists of cellular components, such as cancer-associated fibroblasts (CAFs), pancreatic stellate cells (PSCs), tumor-associated macrophages (TAMs), immune cells, pancreatic cancer cells (PCCs), as well as noncellular elements, including extracellular matrix (ECM) [3]. Reciprocal communication between cells affects the aggressiveness of pancreatic cancer and the effectiveness of cancer therapy by sharing cellular factors that can modulate diverse signaling pathways. In addition, ECM can serve as a barrier to anticancer therapies and as nutrient sources for PCCs and possibly for other cells [3].

Accumulating evidence suggested that extracellular vesicles (EVs), such as exosomes and microvesicles (MVs), can affect various cancer cell properties. For example, the proliferation and migration of PANC-1 cells can be stimulated upon exposure to EVs isolated from serum of patients with pancreatic cancer [4]. In addition, it was recently reported that exosomes shed by CAFs can deliver and supply a variety of metabolites to cancer cells, thus enhancing the proliferation in nutrient-deprived conditions [5].

Moreover, a recent study demonstrated that exosomes derived from pancreatic cancer patients can enhance the proliferation, migration and invasion capacity of PCCs, such as MiaPaCa-2 and AsPC-1 cells [6]. In that study, proteomic analysis of exosomes identified that over 100 proteins are differentially expressed in pancreatic cancer-derived exosomes compared to exosomes from healthy subjects [6]. Overall, these findings clearly indicate the cancer-supporting role of EVs.

Exosomes originated from PCCs can, furthermore, transport cargo molecules to different cell types, ultimately affecting cancer progression. For example, cancer cells can suppress the function of immune cells via their exosomes. Treatment of T lymphocytes with cancer cell-released exosomes gives rise to apoptosis of T cells via activating p38 MAPK-mediated endoplasmic reticulum (ER) stress [7]. In addition, it was suggested that cancer cell-secreted exosomes contribute to the development and survival of monocytic myeloid-derived suppressor cells, possibly via an increase in STAT3 signaling in cultured cells [8]. Another interesting finding is that the direct communications between cancer cells and endothelial cells can take place through exosomes. Exosomes from cancer cells stimulate tube formation and Akt/ERK signaling pathways in endothelial cells, indicating that exosomes function as angiogenesis stimulators [9].

As stated above, EV-based intercellular communication ultimately exerts influence on the biologic features of cancer and cancer-associated cells, and it can prompt cancer aggressiveness, such as angiogenesis and evasion of immune surveillance. Indeed, several EVs inhibitors have been attempted to block the generation and release of EVs and to test their therapeutic benefit for pathologic conditions [10]. This article aims to delineate the significant role of EVs and their cargo molecules in pancreatic cancer. We mainly emphasize recent investigations highlighting the oncogenic function of cargo molecules in association with cancer aggressiveness, such as angiogenesis, metastasis, evasion of immune surveillance, therapeutic resistance, etcetera. We also discuss the cellular components and mechanisms underlying EVs generation, release and uptake in pancreatic cancer to outline the possibility of inhibiting EVs for developing therapeutic strategies to manage pancreatic cancer.

## 2. Effects of EVs and Their Cargo Molecules on Pancreatic Cancer

PCCs can be affected by EVs originated from neighboring cancer cells and other cellular components within the cancer microenvironment. EV-mediated cargo delivery ultimately modulates the diverse properties of PCCs. Several studies uncovered the role of an individual cargo molecule in pancreatic cancer progression, as discussed below.

### 2.1. RNA Cargo in PCC-Derived EVs

#### 2.1.1. MiRNA-23b-3p and miRNA-222

**Figure 1:**
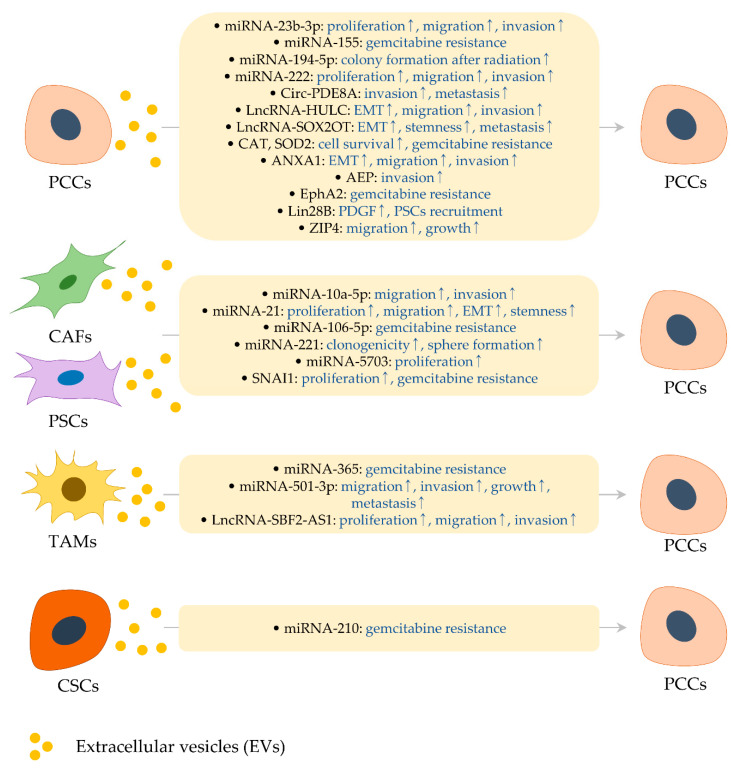
Effects of extracellular vesicle (EV) cargo molecules on the pancreatic cancer cells. Extracellular vesicles from various types of cells harbor cargo molecules (indicated by black letters in rounded rectangles), affecting the biologic properties of pancreatic cancer cells (indicated by blue letters in rounded rectangles). It is described in Section 2 and Table 1.

Recent evidence suggested that high levels of miRNA-23b-3p are detected in exosomes from PANC-1 cells. In this study, it was investigated that exosome-mediated transfer of miRNA-23b-3p can promote the proliferation, migration and invasion of PANC-1 cells [11] (Figure 1 and Table 1).

Owing to the communication between cells via exosomes, miRNA-222 can be transferred from cancer cells to other cancer cells. A recent study revealed that miRNA-222 levels are bountiful in exosomes secreted from PCCs. This miRNA contributes to the augmented proliferation, migration and invasion in exosome-receiving cells in vitro and in vivo. The expression level and localization of p27 (also known as cyclin-dependent kinase inhibitor 1B, CDKN1B) are regulated by miRNA-222 since this miRNA directly targets p27 and protein phosphatase 2 regulatory subunit B alpha (PPP2R2A) [12] (Figure 1 and Table 1). PPP2 is a Ser/Thr phosphatase composed of a catalytic subunit and a constant regulatory subunit, and this enzyme negatively controls cellular processes, such as cell growth, migration and invasion by dephosphorylating Akt and consequently inducing p27 [13,14].

#### 2.1.2. MiRNA-155 and ROS Detoxification Genes

In the case of miRNA-155, it is one of the miRNAs enriched in exosomes that are shed from gemcitabine-resistant cancer cells [15]. Cancer cells also release exosomes with high amounts of miRNA-155, followed by short-term treatment with gemcitabine [16]. It implies a possibility that miRNA-155 may account for the modulation of drug efficacy in exosome-receiving cancer cells. Indeed, miRNA-155 can block the induction of apoptosis, possibly due to the ability of miRNA-155 to target tumor protein p53 inducible nuclear protein 1 (TP53INP1) and deoxycytidine kinase (DCK) genes [15,16] (Figure 1 and Table 1). TP53INP1 is generally known to induce apoptosis by interacting and phosphorylating p53 [17]. DCK is one of the critical intracellular enzymes involved in the generation of an active form of gemcitabine. Therefore, the downregulation of DCK can cause therapeutic resistance to gemcitabine [18,19].

Moreover, exosomes released from gemcitabine-treated cells also harbor transcripts involved in reactive oxygen species (ROS) detoxification, such as catalase (CAT) and superoxide dismutase 2 (SOD2). Delivery of CAT and SOD2 transcripts can reduce ROS levels in exosome-receiving cells, ultimately contributing to gemcitabine resistance [16] (Figure 1 and Table 1).

#### 2.1.3. MiRNA-194-5p

One of the causes of radiotherapy failure is cancer repopulation, which can be promoted by radiotherapy-induced dying cancer cells (RI-DCCs). It was recently identified that exosomes harboring miRNA-194-5p are secreted by RI-DCCs and transferred to other cancer cells. This exosomal miRNA activates DNA damage response, resulting in enhanced cell survival of receiving cells after radiation due to the ability of miRNA-194-5p to regulate target genes, such as E2F transcription factor 3 (E2F3) and high mobility group AT-hook 2 (HMGA2) [20]. In this study, it was also found that miR-196b-5p is enriched in exosomes from RI-DCCs, implying that this miRNA may serve as an exosome-originating survival factor for irradiated cells (Figure 1 and Table 1). Further investigations are required to comprehensively address the function of exosome cargo. Moreover, besides exosomes, RI-DCCs were also demonstrated to release a high amount of prostaglandin E2 (PGE2). Aspirin was identified to impede the secretion of exosomes and PGE2, thereby suppressing cancer repopulation following radiotherapy [20].

#### 2.1.4. Circ-PDE8A

Generally, circular RNAs carry miRNA-binding sequences and serve as miRNA sponges. It has been reported that several circular RNAs are dysregulated in pancreatic cancer and they play an essential role in the progression of pancreatic cancer via sequestering intracellular miRNAs [21,22,23]. Lately, the high expression of circular RNA PDE8A (circ-PDE8A) was identified in exosomes released from PCCs [24]. In this study, it was uncovered that circ-PDE8A inhibits the function of miRNA-338, consequently escalating the levels of metastasis-associated in colon cancer 1 (MACC1), a target of miRNA-338 [24] (Figure 1 and Table 1). Since MACC1 is a positive regulator of c-MET, a receptor for hepatocyte growth factor, circ-PDE8A activates downstream signaling factors, such as Akt and ERK1/2, thereby promoting invasive growth and metastasis of cancer cells [24]. It can be postulated that circ-PDE8A may modulate resistance-related pathways in cancer cells of its origin as well as neighboring cancer cells because c-MET-related signaling pathways are associated with the development of therapeutic resistance in cancer [25].

#### 2.1.5. LncRNAs

Long noncoding RNAs (lncRNAs) are regulatory RNA transcripts and possess the salient features that modulate numerous intracellular signaling pathways in several diseases. They are differentially expressed in various types of cancer and can affect miRNA activity by way of acting as molecular sponges. In addition, the interaction between lncRNAs and chromatin modifiers can modulate gene expression. Moreover, lncRNAs are known to exert influence on post-transcriptional and post-translational events, such as RNA splicing and protein degradation [26,27].

LncRNA-HULC can interact with autophagy related 7 (ATG7) proteins, serving as an oncogenic factor through inhibiting the ATG7-related pathway in ovarian cancer [28]. Additionally, lncRNA-HULC is known to serve as a miRNA-613 sponge, therefore accelerating proliferation and metastasis of colon cancer cells along with the upregulation of rhotekin, a target of miRNA-613 [29]. In pancreatic cancer, it was pointed out that the expression levels of lncRNA-HULC can be stimulated by transforming growth factor-beta (TGFβ). TGFβ treatments downregulate miR-133b, which can interact and degrade lncRNA-HULC. In addition, EV-mediated transfer of lncRNA-HULC into cancer cells promotes proliferation, migration and invasion via positively modulating epithelial-mesenchymal transition (EMT)-promoting elements, such as vimentin and snail [30] (Figure 1 and Table 1).

SRY-Box transcription factor 2 (SOX2), one of the stemness factors, has been demonstrated to aggravate therapeutic resistance and promote invasion and metastasis in several cancer types [25,31]. Recently, it has been demonstrated that lncRNA-SOX2OT positively controls SOX2 expression via sponging the miRNA-200 family, which targets SOX2 transcripts [32,33]. LncRNA-SOX2OT also increases SOX2 levels via demethylating SOX2 transcripts by recruiting alkylated DNA repair protein AlkB homolog 5 (ALKBH5), which specifically demethylates m6A RNA. Therefore, this lncRNA could activate Wnt/β-catenin signaling, conferring resistance to temozolomide in glioblastoma [34]. In pancreatic cancer, lncRNA-SOX2OT is packaged into exosomes and known to be released from highly invasive cancer cells. LncRNA-SOX2OT can promote cancer stemness, EMT, invasion, as well as liver metastasis of circumjacent cancer cells [33] (Figure 1 and Table 1).

### 2.2. Protein Cargo in PCC-Derived EVs

#### 2.2.1. AEP

It has been determined that asparaginyl endopeptidase (AEP, also called legumain) is overexpressed in various cancer types and triggers the EMT process as well as metastasis via stimulating Akt and MAPK signaling pathways [35,36,37]. In pancreatic cancer, it was noted that AEP exists in exosomes and that AEP-harboring exosomes potentiate phosphoinositide 3-kinase (PI3K) signaling pathways, causing enhanced invasion ability of exosome-receiving cells [38] (Figure 1 and Table 1). Intriguingly, patients with chronic pancreatitis, one of the causes of pancreatic cancer, show the high levels of AEP in macrophages [39]. Furthermore, AEP is detected in stroma and endothelial cells in pancreatic cancer tissues [40]. This underlines the possibility that cancer cells can provide AEP to other cell types or vice versa.

#### 2.2.2. ANXA1

It has been demonstrated that cargo proteins from cancer cells of origin can affect various biologic properties of recipient cancer cells. Annexin A1 (ANXA1) is associated with malignant features of pancreatic cancer. ANXA1 can stimulate migration, invasion and metastasis of cancer cells, possibly due to the cytoskeletal remodeling and the activation of signaling pathway related to formyl peptide receptors [41,42]. A recent finding indicated that ANXA1 can be incorporated into EVs. The ANXA1-containing EVs leads to an activation of the EMT process, along with the increased motility of exosome-receiving PCCs [43] (Figure 1 and Table 1). However, another study indicated the opposing role of ANXA1 in PCCs. The resistance to gemcitabine and 5-fluorouracil can be developed in ANXA1-silenced PCCs [44]. These findings suggest that therapeutic targeting of ANXA1 requires careful consideration, especially when combined with anticancer agents. Further investigations will be necessary to screen the effects of ANXA1 silencing on the efficacy of other therapeutic agents.

#### 2.2.3. EphA2

Ephrin type-A receptor 2 (EphA2) is frequently overexpressed in different types of cancer and has been reported to impinge on multiple signaling pathways, including PI3K, Akt and MAPK. In terms of chemoresistance, EphA2 can contribute to therapeutic resistance to several anticancer agents, such as an anti-EGFR tyrosine kinase inhibitor and vemurafenib. Indeed, the inhibition of EphA2 sensitizes cancer cells to anticancer therapies [45,46,47]. In the case of pancreatic cancer, a recent study demonstrated that EphA2 is present in exosomes from gemcitabine-resistant cells [48]. An application of EphA2-carrying exosomes can reshape the phenotype of sensitive cells to gemcitabine-resistant cells [48] (Figure 1 and Table 1). Moreover, since EphA2/TGFβ/COX2 signaling in cancer cells is known to suppress the number of infiltrating T lymphocytes in pancreatic cancer [49], it may be possible that exosome-mediated EphA2 delivery between cancer cells further concurrently influences on cancer microenvironment.

#### 2.2.4. Lin28B

Pancreatic stellate cells (PSCs), one of the resident cell types in pancreatic cancer, support the proliferative and migratory activities, as well as the viability of cancer cells by the secretion of plentiful growth factors, cytokines, extracellular matrix (ECM) components and other factors [50]. In addition, it was demonstrated that PSCs disengaged from the primary location can be found at the metastatic site and that they can be recruited by platelet-derived growth factor (PDGF), a chemotactic factor, secreted from cancer cells. Within this location, PSCs are considered to establish the favorable microenvironment for supporting PCCs [50,51]. The new evidence has shown that exosomes from cancer cells contain Lin-28 homolog B (Lin28B) which can be transmitted to adjacent cancer cells. Lin28B is suggested to positively regulate the levels of PDGF expression through repressing let-7 [52] (Figure 1 and Table 1). Lin28B represses the biogenesis of let-7, which directly targets KRAS [53,54]. In addition, forkhead box M1 (FOXM1), a transcription factor of PDGF, can be activated by KRAS [55,56]. Therefore, it can be inferred that Lin28B may upregulate PDGF levels via the let-7/KRAS/FOXM1 axis.

#### 2.2.5. ZIP4

Zrt- and Irt-like protein 4 (ZIP4) can facilitate the growth of cancer cells and the EMT process via regulating cyclic AMP-responsive element-binding protein 1 (CREB1) and zinc finger E-box binding homeobox 1 (ZEB1), respectively. In addition, the upregulation of ZEB1 can restrict the intracellular concentration of gemcitabine via inhibiting the levels of equilibrative nucleoside transporter 1 (ENT1), an influx transporter of gemcitabine, in pancreatic cancer [57,58]. A recent study indicated that ZIP4 is upregulated in the exosomes secreted from highly malignant cells compared to those from less aggressive cancer cells. In fact, the application of ZIP-4-containing exosomes into cancer cells advances cancer growth in a xenograft mouse model [59] (Figure 1 and Table 1). Based on the function of ZIP-4, it can be also assumed that ZIP-4-harboring exosomes restrict the efficacy of gemcitabine.

### 2.3. RNA Cargo in EVs from PSCs and CAFs

#### 2.3.1. MiRNA-10a-5p

Oncogenic miRNA-10a-5p directly regulates multiple tumor-suppressive genes, namely transcription factor AP-2 gamma (TFAP2C), phosphatase and tensin homolog (PTEN) and cell adhesion molecule L1 like (CHL1) [60,61,62]. Accumulating evidence has been demonstrated that miRNA-10a-5p is overexpressed in pancreatic cancer and advances cell growth, metastasis and chemoresistance [60,63]. Moreover, the expression levels of miRNA-10a-5p were identified to be bountiful in CAF-derived exosomes (Figure 1 and Table 1). Other miRNAs, such as miRNA-92ab-3p, were also copiously detected in exosomes in addition to miRNA-10a-5p [64]. In that study, it was also pointed out that the treatment of CAFs with calcitriol, an active type of Vitamin D, induces vitamin D receptor (VDR)-mediated signaling and remarkably downregulates miRNA-10a-5p levels in exosomes as well as exosome-receiving cancer cells [64]. It provides evidence that VDR activation can lead to anticancer effects on pancreatic cancer partly via downregulating miRNA-10a-5p. Consistent with these observations, it has been shown that VDR is highly expressed in PSCs and CAFs and the activation of VDR can improve the therapeutic response by prompting stromal remodeling [65].

#### 2.3.2. MiRNA-21 and miRNA-221

It was demonstrated that both clonogenicity and sphere formation of PCCs can be enhanced through culturing with PSC- or CAF-derived conditioned media enriched in miRNA-21 and miRNA-221 [66]. Another study also provided evidence that PSC-derived exosomes trigger cell proliferation and migration, together with the upregulation of chemokine (C–X–C motif) ligand 1 and 2 (CXCL1 and CXCL2) and C–C motif chemokine ligand 20 (CCL20) [67]. In this study, miRNA-21 levels were verified to be abundant in exosomes, along with other miRNAs [67] (Figure 1 and Table 1).

#### 2.3.3. MiRNA-106-5p

The treatment of CAFs with gemcitabine leads to the upregulation of oncogenic miRNA-106-5p in CAFs themselves as well as their exosomes [68]. A reduction in miRNA-106-5p levels in these CAF-released exosomes sensitizes PCCs to gemcitabine since this miRNA has an ability to target TP53INP1 in cancer cells [68]. These results indicate that CAF-originated miRNA-106-5p is transported to cancer cells and contributes to gemcitabine resistance (Figure 1 and Table 1). However, miRNA-106-5p can sensitize cholangiocarcinoma cells to 5-fluorouracil by targeting zinc finger and BTB domain containing 7A (ZBTB7A) [69]. It implies that the effects of miRNA-106-5p on anti-cancer agents can be different in a cellular context-dependent manner.

#### 2.3.4. MiRNA-5703

Chemokine-like factor (CKLF)-like MARVEL transmembrane domain-containing (CMTM) family is composed of nine members, which play critical roles in tumorigenesis. For example, CMTM4 has been identified to act as a tumor suppressor by inhibiting cell proliferation, cell cycle and migration in several types of cancer [70,71,72]. Recently, it was demonstrated that exosomes derived from PSCs bear miRNA-5703. This miRNA can assist PSC-originating exosomes in the promotion of cell growth by targeting CMTM4 in PCCs. CMTM4 can negatively regulate cell proliferation via suppressing the PI3K/Akt signaling pathway in pancreatic cancer [73] (Figure 1 and Table 1).

#### 2.3.5. SNAI1

Snail family transcriptional repressor 1 (SNAI1) is one of the EMT-regulating transcription factors and positively regulates cell proliferation and *P*-glycoprotein (*P*-gp) levels, thereby contributing to chemoresistance [25,74]. Exosomes derived from gemcitabine-treated CAFs contain SNAI1 and support the growth and survival of exosome-receiving cancer cells. It ultimately leads to the attenuated response of cancer cells to gemcitabine [75] (Figure 1 and Table 1).

### 2.4. RNA Cargo in EVs from TAMs and NKCs

#### 2.4.1. MiRNA-365

Exosomal miRNAs from TAMs can be responsible for cancer therapeutic resistance. Treatment with gemcitabine markedly upregulates miRNA-365 levels in cancer cells. In addition, this miRNA is existing in exosomes from TAMs and can be delivered into cancer cells, implying that miRNA-365 is one of the potential resistance-related miRNAs [76]. In fact, the knockdown of miRNA-365 in TAMs leads to a reduction in its levels in exosome-receiving cancer cells. It leads to the augmented apoptosis induction of cancer cells following the treatment of gemcitabine. Furthermore, the enhancement of gemcitabine sensitivity can be achieved by inhibiting miRNA-365 levels in vivo. A mechanism underlying miRNA-365-mediated chemoresistance indicated that this miRNA acts as a resistance factor by elevating cytidine deaminase enzymes, which inactivate gemcitabine [76] (Figure 1 and Table 1).

#### 2.4.2. MiRNA-501-3p

TGFβ receptor 3 (TGFBR3) is generally considered as a tumor-suppressor and restrains migration and metastasis in several types of cancer, including pancreatic cancer [77,78,79]. In line with this, the expression of TGFBR3 is downregulated in cancer tissues compared to the normal tissues [77]. Recently, it was noted that TAMs play a part in downregulating TGFBR3 expression in pancreatic cancer [80]. TAM-derived exosomes advance migration, invasion and metastasis of cancer cells in vitro and in vivo and it can be mediated by exosomal miRNA-501-3p, which certainly targets TGFBR3. Indeed, overexpression of TGFBR3 can reverse the effects of TAM-derived exosomes and miRNA-501-3p on cancer cells [80] (Figure 1 and Table 1).

#### 2.4.3. LncRNA-SBF2-AS1

Similarly, it was revealed that exosomes originated from TAMs carry lncRNA-SBF2-AS1, which can interfere with miRNA function [81]. This lncRNA from TAMs affects the progression of cancer cells by disturbing miRNA–target gene interactions. Actually, exosomal transfer of lncRNA-SBF2-AS1 into cancer cells represses the activity of miRNA-122-5p, thereby upregulating X-linked inhibitor of apoptosis protein (XIAP), a pro-survival factor. In a xenograft mouse model of pancreatic cancer, the application of exosomes from TAMs lacking lncRNA-SBF2-AS1 retards the growth of cancer [81] (Figure 1 and Table 1). On the basis of other investigations, it can be presumed that lncRNA-SBF2-AS1 orchestrates various signaling pathways to positively regulate the progression of cancer since that miRNA-122-5p can target other genes, such as cyclin G1 (CCNG1) [82], and that lncRNA-SBF2-AS1 is known to sponge other miRNAs [83].

### 2.5. RNA Cargo in EVs from CSCs

#### MiRNA-210

Owing to several intrinsic features of cancer stem cells (CSCs), they are generally resistant to cancer treatments. For example, high levels of ATP-binding cassette transporters (ABC transporters) are detected in CSCs and responsible for chemoresistance [25,84]. Moreover, it has been underscored that exosomes from CSCs can reprogram non-CSCs into CSCs [85]. Recently, another study provided specific evidence that miRNA-210 is elevated in exosomes released from gemcitabine-resistant CSCs, and this miRNA can confer gemcitabine resistance in recipient cells, along with the increased levels of resistance-related genes, such as ABCB1 (also known as multidrug resistance protein 1 (MDR1) and *P*-glycoprotein (*P*-gp)) (Figure 1 and Table 1). It remains to be precisely determined how this miRNA affects the sensitivity of cancer cells to gemcitabine. However, other studies suggested that miRNA-210 can raise the levels of CSC factors and maintain self-renewal capacity [86,87].

**Table 1:** Cargo molecules in EVs from various types of cells and effects of cargo molecules on pancreatic cancer cells (PCCs).

Cargo	Source of EVs	Type of Study	Major Function of Cargo Molecules	Ref.
miRNAs				
miRNA-10a-5p	CAFs isolated from human pancreatic cancer tissues	In vitro	Support the aggressiveness of PANC-1 and SW1990 cells	[64]
miRNA-21	PSCs (human PSC21-S/T cell line), CAFs (human CAF-19 cell line)	In vitro	Reinforce the proliferation, migration and EMT process of PANC-1 and SUIT-2 cells; augment clonogenicity and sphere formation of Colo-357 cells	[66,67]
miRNA-23b-3p	PCCs (human PANC-1 cells)	In vitro	Increase the proliferation, migration and invasion of PANC-1 cells	[11]
miRNA-106-5p	CAFs isolated from human pancreatic cancer tissues	In vitro	Confer gemcitabine resistance in AsPC-1 cells	[68]
miRNA-155	PCCs (gemcitabine-treated human MIAPaCa-2 and Colo-357 cells, gemcitabine-resistant human PANC-1 cells)	In vitro, In vivo	Inhibit gemcitabine-induced apoptosis in MIAPaCa-2 and Colo-357 cells in vitro; confer gemcitabine resistance in PANC-1 cells in vivo	[15,16]
miRNA-194-5p	Irradiated human PANC-1 and SW1990 cells	In vitro, In vivo	Augment the survival of SW1990 cells following radiation in vitro	[20]
miRNA-210	CSCs derived from gemcitabine-resistant human BxPC-3 cells	In vitro, In vivo	Inhibit gemcitabine-induced apoptosis in BxPC-3 and PANC-1 cells in vitro; confer gemcitabine resistance in BxPC-3 cells in vivo	[88]
miRNA-221	CAFs (human CAF-19 cell line), PSCs isolated from human pancreatic cancer tissues	In vitro	Stimulate the clonogenicity and sphere formation of Colo-357 cells	[66]
miRNA-222	PCCs (human Hs 766 T-L3 cells)	In vitro, In vivo	Enhance the proliferation, migration and invasion of CAPAN-1 and Hs 766 T-L3 cells in vitro; promote cancer progression in vivo	[12]
miRNA-365	TAMs (M2-polarized murine peritoneal macrophages)	In vitro, In vivo	Attenuate the gemcitabine efficacy in K989 murine cells	[76]
miRNA-501-3p	TAMs (M2-polarized human THP-1 cells)	In vitro, In vivo	Enhance the migration and invasion of PANC-1 and BxPC-3 cells in vitro; promote cancer growth and metastasis in vivo	[80]
miRNA-5703	PSCs isolated from human pancreatic cancer tissues	In vitro	Promote the proliferation of Patu8988 and T3M4 cells	[73]
Other non-coding RNAs				
Circ-PDE8A	PCCs (human Hs 766 T-L2 cells)	In vitro, In vivo	Facilitate the invasion of BxPC-3 and CAPAN-1 cells in vitro; enhance liver metastasis in vivo	[24]
LncRNA-HULC	PCCs (human PANC-1 cells)	In vitro, In vivo	Trigger migration, invasion and EMT process in PANC-1 and MIAPaCa-2 cells in vitro; promote cancer progression in vivo	[30]
LncRNA-SBF2-AS1	TAMs (M2-polarized human THP-1 cells)	In vitro, In vivo	Enhance the proliferation, migration and invasion of PANC-1 cells in vitro; force the tumorigenic ability of PANC-1 cells in vivo	[81]
LncRNA-SOX2OT	PCCs (human Hs 766 T and Hs 766 T-L2 cells)	In vitro, In vivo	Promote EMT and stemness in Hs 766 T cells in vitro; trigger EMT, stemness and metastasis in vivo	[33]
mRNAs				
CAT and SOD2	PCCs (gemcitabine-treated human MIAPaCa-2 and Colo-357 cells)	In vitro	Protect cell death induced by ROS in gemcitabine-treated MIAPaCa-2 cells	[16]
SNAI1	CAFs isolated from human pancreatic cancer tissues	In vitro	Promote the proliferation and gemcitabine resistance in AsPC-1 cells	[75]
Proteins				
AEP	PCCs (human BxPC-3 cells)	In vitro	Aggravate the invasion ability of BxPC-3 and AsPC-1 cells	[38]
ANXA1	PCCs (human MIAPaCa-2 cells)	In vitro	Facilitate the EMT, migration and invasion in MIAPaCa-2 cells	[43]
EphA2	PCCs (gemcitabine-resistant human PANC-1 cells)	In vitro	Develop gemcitabine resistance in MIAPaCa-2 and BxPC-3 cells	[48]
Lin28B	PCCs (human PANC-1 and MIAPaCa-2 cells)	In vitro, In vivo	Increase the levels of PDGF in PANC-1 and MIAPaCa-2 cells, ultimately enhancing PSCs recruitment to the metastatic site	[52]
ZIP4	PCCs (hamster PC-1.0 cells)	In vitro, In vivo	Promote the proliferation and migration of PC-1.0 cells in vitro; enhance the growth of cancer in vivo	[59]

**Abbreviations:** AEP: Asparaginyl endopeptidase; ANXA1: Annexin A1; CAFs: Cancer-associated fibroblasts; CAT: Catalase; CSCs: Cancer stem cells; EMT: Epithelial-mesenchymal transition; EphA2: Ephrin type-A receptor 2; EVs: Extracellular vesicles; Lin28B: Lin-28 homolog B; MiRNA: MicroRNA; PDGF: Platelet-derived growth factor; PSCs: Pancreatic stellate cells; ROS: Reactive oxygen species; SNAI1: Snail family transcriptional repressor 1; SOD2: Superoxide dismutase 2; TAMs: Tumor-associated macrophages; ZIP4: Zrt- and Irt-like protein 4.

## 3. Effects of PCC-Derived EVs on the Cellular Components in Pancreatic Cancer Microenvironment and Metastatic Site

PCCs can affect other neighboring or distant cells by transferring their EVs. The role of an individual cargo in the regulation of the fate of other cell types was underscored, as stated below.

### 3.1. Regulation of Endothelial Cells by PCC-Derived EVs

#### 3.1.1. MiRNA-27a

**Figure 2:**
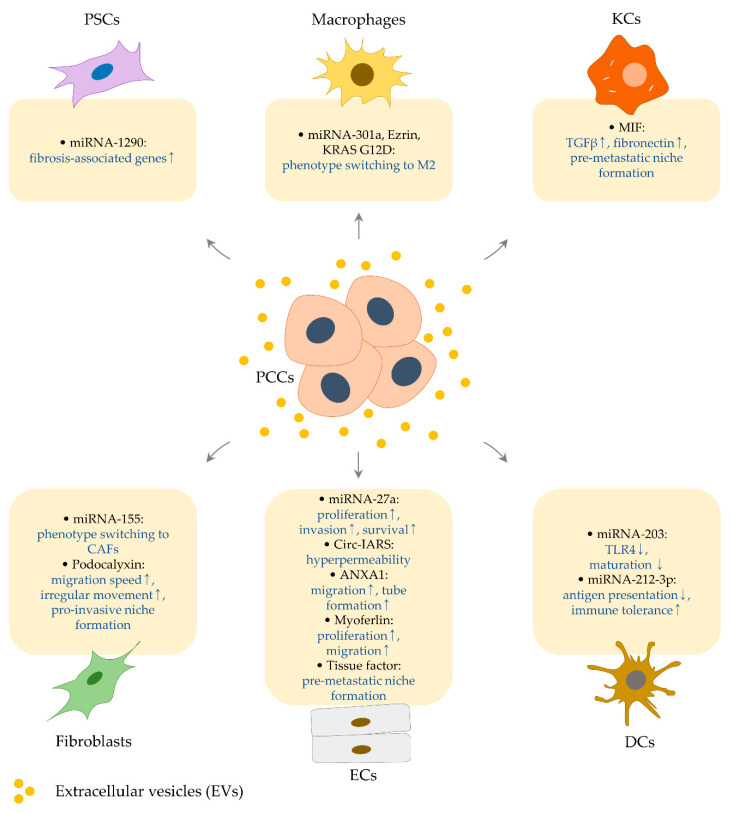
Effects of PCC-derived EVs and their cargo molecules on the other types of cells. Extracellular vesicles from pancreatic cancer cells retain various cargo molecules (indicated by black letters in rounded rectangles), controlling the biologic properties of adjacent or distant cells (indicated by blue letters in rounded rectangles). It is described in Section 3 and Table 2.

Several studies have demonstrated that miRNA-27a negatively regulates endogenous expression of anti-angiogenic factors, such as semaphorin 6A (SEMA6A) and SMAD family member 4 (SMAD4) in endothelial cells (ECs) [89,90]. Moreover, a recent article indicated that this pro-angiogenic miRNA is expressed in PCCs themselves as well as their exosomes and transported into ECs via exosomes. Transferred miRNA-27a can positively regulate the proliferation and invasion of ECs feasibly through targeting BTG antiproliferation factor 2 (BTG2) [91] (Figure 2 and Table 2). Indeed, BTG2 has been generally known to negatively regulate cell cycle/cell proliferation and induce apoptosis in several cell types [92,93,94].

#### 3.1.2. Circ-IARS

Endothelial hyperpermeability caused by the loss of barrier integrity is a crucial step for metastasis events. It has been realized that the permeability of vessels and cancer metastasis can be advanced by multiple factors released from cancer cells, such as vascular endothelial growth factor (VEGF) and secreted protein acidic and cysteine-rich (SPARC) [95,96]. In the case of SPARC, it can induce endothelial hyperpermeability via interacting with vascular cell adhesion molecule 1 (VCAM1) and activating ROS-p38 MAPK signaling pathways [95]. Besides these instances, it was reported that a non-coding RNA also participates in the regulation of endothelial barrier integrity. Exosomal circ-IARS can be released to the outside of cancer cells and transported into ECs, eventually eliciting endothelial hyperpermeability [97]. In ECs, circ-IARS downregulates the levels of tight junction protein 1 (TJP1, also known as zona occludens (ZO-1)) through restraining the function of miRNA-122 that targets Ras homolog family member A (RhoA) [97] (Figure 2 and Table 2). Activation of RhoA can obstruct endothelial barrier integrity by promoting the formation of stress fibers [98].

#### 3.1.3. ANXA1

ANXA1 is associated with multiple cellular events. For example, ANXA1 can act as a mediator of VEGF effects on ECs, thereby stimulating the migration of ECs and regulating lamellipodia formation. Knockdown of ANXA1 can abrogate VEGF-induced migration and tube formation of ECs [99,100]. As stated in Section 2.2.2, ANXA1 can be incorporated in PCC-derived EVs and modulate the motility of cancer cells. Beyond this, it was unequivocally addressed that ANXA1 in PCC-derived EVs can activate ECs, and it was ascertained by monitoring proliferation, migration, invasion and tube formation of endothelial cells [43] (Figure 2 and Table 2).

#### 3.1.4. Myoferlin

Myoferlin regulates multiple biologic events, such as endocytosis and membrane repair/fusion. In pancreatic cancer cells, myoferlin is noticed to maintain the structure and activity of mitochondria, thereby enhancing the growth and migration of cancer cells [101,102]. In addition, myoferlin can protect the Cbl-induced proteasomal degradation of VEGF receptor 2 (VEGFR2) in ECs [103]. In terms of cell–cell communication, myoferlin is one of the components of PCC-derived exosomes and can be carried to ECs, thereby favorably affecting the proliferation and migration of ECs. Likewise, myoferlin-deficient exosomes are unable to support the growth and migration of ECs [104] (Figure 2 and Table 2).

#### 3.1.5. Tissue Factor

Tissue factor (TF) involved in the generation of coagulation factor Xa (FXa) and thrombin is aberrantly expressed in pancreatic cancer and contributes to venous thromboembolism [105]. In addition, restraining of its function retards cancer growth and metastasis [106]. Protease-activated receptors (PAR) and their downstream signaling factors, such as RhoA, are triggered by thrombin and FXa and responsible for the stimulation of endothelial hyperpermeability and metastasis [107,108]. The new findings suggested that TF in PCC-derived EVs activates ECs and converts them into inflammatory phenotypes via the FXa-PAR1 axis. Immunological and pharmacological inhibition of TF, FXa, and PAR1 attenuates the response of ECs to TF-harboring EVs, implying the possibility that TF can facilitate the formation of pre-metastatic niche and metastasis [109] (Figure 2 and Table 2).

### 3.2. Regulation of Fibroblasts and Stellate Cells by PCC-Derived EVs

#### 3.2.1. MiRNA-155

In addition to the role of miRNA-155 in cancer cells (Section 2.1.2), this miRNA is associated with the reprogramming of fibroblasts. PCC-derived EVs contain miRNA-155 and can deliver miRNA-155 into normal fibroblasts. Treatments with EVs turn the feature of normal fibroblasts into that of CAFs. By targeting TP53INP1 in fibroblasts, miRNA-155 is deemed to cause this conversion [110] (Figure 2 and Table 2). In terms of TP53INP1, it was demonstrated that the blocking of TP53INP1 activity can activate fibroblasts and increase in CAF markers, such as fibroblast growth factor 2 (FGF2) and alpha-smooth muscle actin (αSMA) [111].

#### 3.2.2. Podocalyxin

Mutation of tumor protein p53 (TP53) is regarded as a watershed in the initiation and progression of cancer and aggravates the invasion, metastasis, as well as chemoresistance [112,113]. Mutant p53 also affects the ECM organization. For example, mutant p53 has the potential to persistently activate Janus kinase (JAK)/signal transducer and activator of transcription 3 (STAT3) signaling, hence stimulating desmoplasia [114]. Additionally, it was revealed that mutant p53 can suppress the amount of podocalyxin in exosomes [115]. Treatment of fibroblasts with exosomes from mutant p53-expressing cancer cells can induce the enhanced speed of migration and erratic movements of fibroblasts, as well as the modulation of ECM organization, thereby contributing to the generation of pro-invasive niches (Figure 2 and Table 2). The knockdown of mutant p53 reverses these effects of exosomes on fibroblasts, along with an increase in podocalyxin levels [115].

#### 3.2.3. MiRNA-1290

PSCs can be activated by various factors, such as TGFβ and FGFs and are accountable for the imbalanced production and degradation of the extracellular matrix components, ultimately leading to the extensive fibrotic microenvironment [116,117]. This alteration of the cancer microenvironment plays a key role in disease progression as well as metastasis [116]. A recent study implies that PCC-derived exosomes activate the proliferation and migration of PSCs, as well as the induction of fibrosis-associated genes, such as collagen type V alpha 1 [118]. In this study, it was also found that miRNA-1290, a profibrogenic gene-regulating miRNA, is increased in PSCs following co-culture with PANC-1 cells. It demonstrates that miRNA-1290 can be transferred into PSCs via exosomes (Figure 2 and Table 2). In fact, miRNA-1290 is one of the abundant miRNAs in PCC-derived exosomes [119].

### 3.3. Effects of PCC-derived EVs on Kupffer Cells

#### MIF

Pancreatic cancer frequently metastasizes to the liver. It was recently demonstrated that cancer metastasis is potentially stimulated by PCC-derived exosomes, which can be hugely engulfed by Kupffer cells (KCs) [120]. In this work, macrophage migration inhibitory factor (MIF) was identified to be distinctly present in exosomes. Transmitted MIF can induce TGFβ in KCs, which in turn contributes to creating a pre-metastatic niche via activating the production of fibronectin from hepatic stellate cells. It ultimately increases the burden of metastatic cancer [120] (Figure 2 and Table 2). In another study, MIF was also demonstrated to restrict nuclear receptor subfamily 3 group C member 2 (NR3C2), which negatively regulates EMT-promoting factors in cancer cells [121]. These findings indicate that MIF can play a pivotal role in the metastatic cascade both at primary cancer site and metastatic lesion.

### 3.4. Effects of PCC-Derived EVs on Macrophages

#### 3.4.1. MiRNA-301a

From the perspective of cancer, hypoxia, one of the hallmarks of cancer microenvironment, is conductive to boost survival of cancer cells, metastasis and therapeutic resistance. For example, the release of EVs is elevated by oxygen deprivation, and EVs support the survival of cancer cells [122]. In addition, hypoxia is responsible for the phenotype switching of macrophages from M1 TAMs to M2 TAMs via oncostatin M and eotaxin, which are cytokines released from hypoxic cells. M2 TAMs are, in turn, responsible for the induction of angiogenesis, EMT, therapeutic resistance and immune suppression in cancer [123,124]. Moreover, phenotype conversion from M1 TAMs to M2 TAMs can be controlled by miRNA-301a packaged in hypoxic cancer cell-derived exosomes [125] (Figure 2 and Table 2). Macrophage polarization can be resulted from the activation of PI3K gamma by exosomal miRNA-301a, which targets PTEN [125].

#### 3.4.2. Ezrin

Ezrin has been proposed to stimulate proliferation, invasion and EMT progression by activating the PI3K/Akt signaling pathway in pancreatic cancer [126]. In addition, Ezrin was found to be activated in pancreatic CSCs, enhancing the colony-forming ability [127]. Recently, it was demonstrated that Ezrin is incorporated in PCC-derived EVs and transferred to macrophages, where Ezrin polarizes macrophages into M2 phenotype. By contrast, EVs from Ezrin-depleted PCCs reduce the number of macrophages with M2 markers, such as CD163 [128] (Figure 2 and Table 2).

#### 3.4.3. KRAS G12D

Kirsten rat sarcoma 2 viral oncogene homolog (KRAS) makes a critical contribution to the initiation and progression of pancreatic cancer via affecting diverse cellular events and G12D is one of notable missense mutations, constitutively activating KRAS in cancer cells [129]. Although KRAS and its effectors (e.g., PI3K, ERK, STAT3) are excellent therapeutic targets, it is still essential to overcome therapeutic resistance to their inhibitors. Recently, it was underscored that KRAS G12D is relayed from cancer cells to macrophages, indicating that KRAS G12D affects the cancer microenvironment [130]. In particular, exosomes bearing KRAS G12D are released from cancer cells upon ferroptotic cell death, a type of autophagic cell death induced by oxidative stress. These exosomes can be internalized into macrophages via advanced glycosylation end-product specific receptor (AGER, also known as RAGE). In macrophages, KRAS G12D activates STAT3-mediated fatty acid oxidation, ultimately leading to the development of M2 TAMs [130] (Figure 2 and Table 2). It suggests that targeting the delivery of KRAS between cells is also required to improve therapeutic response.

### 3.5. Effects of PCC-Derived EVs on Dendritic Cells

#### 3.5.1. MiRNA-203

Toll-like receptor 4 (TLR4) is a pivotal mediator of dendritic cell activation via recognizing danger-associated molecular patterns (DAMP), and it is critical for the process and presentation of tumor antigens. Activated dendritic cells, in turn, trigger anticancer responses of T-cells [131]. However, the activity of dendritic cells (DCs) can be weakened within the cancer microenvironment. For example, DCs can be inactivated by TAM-derived interleukin-10 (IL-10), eventually dampening the anticancer activities of T-cells [132]. Moreover, cancer cells can render negative impacts on DCs. A recent study indicated that PCC-derived exosomes carry miRNA-203 and can be delivered into DCs [133]. Exosome-receiving DCs show the downregulation of TLR4, and it can be due to the targeting of TLR4 by miRNA-203. Moreover, lowering TLR4 levels mediated by miRNA-203 can cause a reduction of the levels of cytokines, such as IL-12 and tumor necrosis factor α (TNFα), which is capable of regulating cellular immunity and DCs maturation, respectively [133] (Figure 2 and Table 2).

#### 3.5.2. MiRNA-212-3p

In a similar manner, PCC-derived exosomes can deliver miRNA-212-3p into DCs and exerts an influence on immune surveillance [134] (Figure 2 and Table 2). A reduction in the levels of major histocompatibility complex class II (MHC class II) can be observed in DCs following exosome exposure. Based on the functional identification of miRNA-212-3p, it was confirmed that miRNA-212-3p directly targets regulatory factor X associated protein (RFXAP) [134], which is a novel transcription factor of MHC class II genes [135].

**Table 2:** Cargo molecules in PCC-derived EVs and their influences on the other cell types in cancer microenvironment and metastatic lesion.

Cargo	Source of EVs	Type of Study	Major Function of Cargo Molecules	Ref.
miRNAs				
miRNA-27a	PCCs (human PANC-1 cells)	In vitro, In vivo	Enhance the proliferation, invasion and survival of human endothelial cells in vitro; promote cancer growth and angiogenesis in vivo	[91]
miRNA-155	PCCs (human BxPC-3 and SW1990 cells)	In vitro	Participate in the conversion from primary mouse fibroblasts to CAFs phenotypes	[110]
miRNA-203	PCCs (human PANC-1 cells)	In vitro	Interrupt the maturation of human dendritic cells	[133]
miRNA-212-3p	PCCs (human PANC-1 cells)	In vitro	Repress the levels of MHC class II in human dendritic cells	[134]
miRNA-301a	Hypoxic PCCs (human PANC-1 cells)	In vitro, In vivo	Convert human bone marrow–derived macrophages into M2 types in vitro; facilitate lung metastasis in vivo	[125]
miRNA-1290	PCCs (human PANC-1 cells)	In vitro	Activate human primary stellate cells; induce fibrogenic genes	[118]
Non-coding RNA				
Circ-IARS	PCCs (human Hs 766 T and Hs 766 T-L2 cells)	In vitro, In vivo	Disrupt the barrier integrity of human endothelial cells in vitro; promote invasion and metastasis in vivo	[97]
Proteins				
ANXA1	PCCs (human MIAPaCa-2 cells)	In vitro	Mediate VEGF-induced migration and formation of the tube structure in human endothelial cells	[43]
Ezrin	PCCs (PC080 and PC084 cells derived from human pancreatic cancer tissues)	In vitro, In vivo	Promote M2 polarization of THP-1/U937-derived macrophages in vitro; facilitate liver metastasis along with a high M2/M1 ratio in vivo	[128]
KRAS G12D	Ferroptotic dying PCCs (human PANC-1 and AsPC-1 cells, primary PCCs from human pancreatic cancer tissues)	In vitro, In vivo	Promote M2 polarization of human mononuclear cell-derived macrophages in vitro; macrophage-mediated cancer growth is retarded by blocking KRAS G12D release and uptake in vivo	[130]
MIF	PCCs (murine PAN02 cells)	In vitro, In vivo	Activate human Kupffer cells in vitro; enhance the formation of liver pre-metastatic niche in vivo	[120]
Myoferlin	PCCs (human BxPC-3 cells)	In vitro	Enhance the proliferation and migration of human endothelial cells	[104]
Podocalyxin	PCCs isolated from human pancreatic cancer tissues	In vitro	Contribute to the generation of pro-invasive niche via regulating the migration of immortalized human dermal fibroblasts	[115]
Tissue Factor	PCCs (human BxPC-3 and CAPAN-1 cells)	In vitro	Activate human endothelial cells by upregulating E-selectin and IL-8 levels in a PAR-1 dependent manner	[109]

**Abbreviations:** ANXA1: Annexin A1; CAFs: Cancer-associated fibroblasts; EVs: Extracellular vesicles; IL-8: Interleukin-8; MHC class II: Major histocompatibility complex class II; MIF: Macrophage migration inhibitory factor; MiRNA: MicroRNA; PAR-1: Protease-activated receptor 1; PCCs: Pancreatic cancer cells; VEGF: Vascular endothelial growth factor.

## 4. Interference with EV-Based Conversation between Cells: Possibilities for Pancreatic Cancer Therapy

Cellular factors and events can modulate the biosynthesis and itinerary of EVs, and it can be modulated by pharmacological or genetic approaches.

### 4.1. Cellular Factors Affecting EVs Biogenesis and Secretion

#### 4.1.1. ANXA1

It was investigated that the knockdown of ANXA1 diminishes the amounts of secreted EVs, particularly exosomes, in pancreatic cancer cells, indicating that ANXA1 positively regulates exosome biosynthesis [43]. ANXA1 is known to create the membrane contact sites and inward vesiculation, which are required for the formation of intraluminal vesicles in multivesicular bodies (MVBs) [136,137]. In addition, the affinity between ANXA1 and the cell membrane can be enhanced by ceramide [138]. Thus, the coordinated regulation of ANXA1 and sphingomyelin phosphodiesterase 3 (SMPD3) may promote exosome biogenesis in pancreatic cancer. The role of SMPD3 is demonstrated in Section 4.1.6.

#### 4.1.2. GIPC

Intracellular events, such as endocytosis and receptor clustering, can be modulated by GIPC PDZ domain-containing family member 1 (GIPC, also known as synectin). It has been shown that GIPC is highly expressed in pancreatic cancer tissues compared to normal tissues. Inhibition of GIPC shows anticancer effects on pancreatic cancer in vitro and in vivo, together with a reduction in the levels of insulin-like growth factor 1 receptor (IGF1R) [139,140,141].

However, it was recently demonstrated that knockdown of GIPC can lead to an increase in exosome secretion by inducing the levels of factors involved in the machinery of exosome biosynthesis, namely ALG-2 interacting protein X (ALIX), tumor susceptibility gene 101 (TSG101) and charged multivesicular body protein 4B (CHMP4B) [142]. Although further investigations are needed, it indicates the possibility that the GIPC-depleted cells may send exosomes to neighboring cells to support their survival in the same way that RI-DCCs transfer pro-survival factor(s) to other cells (Section 2.1.3).

#### 4.1.3. PAFR

As stated in Table 1, EVs can be released from gemcitabine-resistant cells or -treated cells and confer neighboring cells resistant to gemcitabine. Recently, the importance of platelet-activating factor receptors (PAFR) in the release of EVs was underscored. It was observed that gemcitabine-induced release of EVs can be blocked by WEB2086, a PAFR antagonist, in PAFR-expressing cancer cells, but not in PAFR-negative cancer cells [143]. In addition, treatments of imipramine, a SMPD3 inhibitor, also impede EVs secretion. Moreover, both ERK1/2- and p38-inhibitors efficiently inhibit the secretion of EVs, suggesting that ERK1/2 and p38 signaling pathways play a role in PAFR-mediated EVs release [143]. It proposes that the combination of gemcitabine with EV-secretion blockers can be considered as an attractive therapeutic strategy for pancreatic cancer therapy.

#### 4.1.4. PAR2

As described in Section 3.1.5., PCC-derived EVs harbor TF, which activates endothelial cells via activating PAR1. Recently, it was uncovered that the secretion of TF-bearing EVs is heightened by PAR2 activation in cancer cells [144]. PAR2 is activated by TF/factor VIIa (fVIIa) complex, which initiates coagulation protease cascade. In this study, it was shown that treatments of cancer cells with apixaban, an anticoagulant, resulted in the downregulation of the release of TF-bearing EVs, together with the reduction of cell proliferation. Apixaban binds to and inactivates fVIIa, thereby preventing PAR2 activation [144].

#### 4.1.5. RAB27

RAB27 subfamily is composed of RAB27A and RAB27B and acts as a regulator of vesicular transport. In particular, this subfamily participates in the secretion of exosomes by mediating the docking of MVBs at the cell membrane. Other RAB proteins, such as RAB9, are also identified to positively regulate exosome secretion [145].

In pancreatic cancer, overexpression of RAB27 is positively correlated with poor prognosis [146,147]. Downregulation of RAB27A/B attenuates proliferation/invasion and also leads to the enhanced efficacy of cisplatin with an induction of apoptosis in pancreatic cancer cells [148]. Moreover, knockdown of RAB27B diminishes the amounts of secreted exosomes, activates caspase 3/7 and sensitizes cells toward gemcitabine [15].

#### 4.1.6. SMPD3

SMPD3 (also known as neutral sphingomyelinase (nSMase2)) catalyzes the generation of ceramide via the sphingomyelin hydrolysis. Ceramide is a class of sphingolipid that triggers exosome formation in MVBs [149,150]. Indeed, it has been demonstrated that the inhibition of SMPD enzymes effectively reduces the exosome-mediated progression of various cancer types [151].

In pancreatic cancer, the treatment of cancer cells and CAFs with GW4869, a SMPD3 inhibitor, interrupts exosome secretion, resulting in the impediment of angiogenesis and survival of cancer cells [75,91]. In addition, it was suggested that GW4869 effectively restricts cancer repopulation caused by RI-DCC-derived exosomes in vivo [20].

It has been demonstrated that the effects of blocking of SMPD3 using GW4869 on exosome secretion can be dependent on the levels of phosphatidylserine and the type of cells [152]. In addition, there is a possibility that the inhibition of SMPD3 blocks the cytotoxicity of tumor necrosis factor (TNF) in cancer cells [153]. Therefore, further research is required to screen the efficacy of GW4869.

#### 4.1.7. MiRNA-155 and lncRNA-PVT1

The fate of exosomes can also be modulated by non-coding RNAs. For example, the overexpression or knockdown of miRNA-155 elevates or drops the amounts of secreted exosomes, respectively. It implies the feasibility that miRNA-155 inactivates RAB proteins via directly or indirectly regulating GTPase-activating proteins [15].

LncRNA-PVT1 is highly expressed in several cancers and associated with poor prognosis. This lncRNA is recognized to enhance growth, migration, invasion and angiogenesis via activating several oncogenic factors, such as beta-catenin, hexokinase 2 and STAT3 [154,155,156]. LncRNA-PVT1 also acts as an anti-apoptotic factor and contributes to 5-fluorouracil Resistance [157]. There is consistent evidence that lncRNA-PVT1 stimulates the proliferation, migration, cytoprotective autophagy by inactivating miRNAs, such as miRNA-20a and miRNA-448 in pancreatic cancer [158,159].

Of particular interest is the role of lncRNA-PVT1 in the modulation of exosome secretion in pancreatic cancer. LncRNA-PVT1 was identified to facilitate the fusion of MVBs with the cell membrane and their secretion by regulating the activity of YKT6 V-SNARE Homolog (YKT6), the colocalization of YKT6 with vesicle-associated membrane protein 3 (VAMP3) and the expression levels of RAB7 [160]. RAB7 is one of the regulators of the transportation and docking processes of MVBs. Soluble N-ethylmaleimide-sensitive factor attachment protein receptors (SNAREs) can drive the exosome fusion events in cells, and YKT6 and VAMP3 are the principal members of SNAREs [161,162].

#### 4.1.8. Other Possible Factors and Their Inhibitors

Several pharmacological compounds, such as manumycin A, tipifarnib, Y27632 and calpeptin, are reported to block exosome generation and secretion. Manumycin A is a farnesyl transferase inhibitor and attenuates the levels of ALIX and RAB27A via inhibiting ERK activity [163]. Tipifarnib also inhibits farnesyl transferase and suppresses ALIX, RAB27A and SMPD3 [164]. In addition, Y27632 and calpeptin are known to repress Rho associated coiled-coil containing protein kinase (ROCK) and calpain, respectively, and they can block the generation and release of exosomes [165].

To our knowledge, there is no direct investigation on whether these compounds regulate exosome biogenesis and secretion in pancreatic cancer. Nonetheless, numerous preclinical studies show their anticancer effects on pancreatic cancer. For example, treatments of manumycin A reduce the growth and invasion of pancreatic cancer cells [166]. Tipifarnib shows effective combinatorial anticancer activities with atorvastatin and celecoxib in vitro and in vivo [167]. Y27632 and calpeptin can suppress the migration and invasion of pancreatic cancer cells [168,169]. Further studies are required to evaluate the effects of these compounds on exosome-mediated connections between diverse cell types in pancreatic cancer.

### 4.2. Regulation of EVs Uptake

#### 4.2.1. AGER

AGER belongs to the immunoglobulin superfamily and is expressed in various cell types, including macrophages. Multiple ligands can bind to AGER and activate proinflammatory signaling pathways. It was also demonstrated that high mobility group box 1 (HMGB1) can bind to and activate AGER in macrophages, strengthening the activity of M2 TAMs [170]. Moreover, PCC-derived exosomes can be internalized into macrophages through AGER. The uptake of KRAS G12D-bearing exosomes can be blocked by anti-AGER antibodies. In addition, the knockdown of AGER achieves the same outcomes (also see Section 3.4.3.) [130].

#### 4.2.2. ANXA6

ANXA6 is incorporated in CAF-derived EVs and forms a complex with other proteins, including LDL receptor-related protein 1 (LRP1) and thrombospondin 1 (TSP1). These CAF-originating EVs can enhance the aggressiveness of PCCs, such as migration and invasion. Although the precise role of ANXA6 is undisclosed, EVs from ANXA6-silencing CAFs significantly lose their ability to enter the recipient cells, implying that ANXA6 or ANXA6-containing complex is responsible for internalization of EVs.

#### 4.2.3. TSPAN8

Tetraspanin 8 (TSPAN8), a membrane glycoprotein, is known to be highly expressed in pancreatic cancer and contributes to the increase in migration and angiogenesis [171]. Concerning exosomes, TSPAN8 as well as other tetraspanins (e.g., CD9 and CD63) are classical exosome surface markers, and TSPAN8 is also implicated in exosome uptake [172,173]. It was recently noted that the expression levels of TSPAN8 are positively regulated by CD44v6 and that exosomes from CD44v6-depleted cancer cells are scarcely engulfed by cells [173].

#### 4.2.4. Dynamin-Dependent Endocytosis

Dynamin 2 is a multifunctional factor that participates in various cellular processes, such as invasion and membrane scission. Dynamin 2 is overexpressed in pancreatic cancer and associated with poor survival. In addition, dynamin 2 can potentiate the invasion of PCCs via interacting with α-actinin 4 and stabilizing Vav guanine nucleotide exchange factor 1 (VAV1) [174,175].

Moreover, the entry of exosomes into the cells is regulated by dynamin-dependent endocytosis. It was shown that the treatment of endothelial cells with dynasore, a reversible inhibitor of dynamin 1/2, prevents the tube formation prompted by PCC-derived exosomes [9]. Dynasore was also identified to block the activation of ERK1/2 in exosome-receiving cells through silencing the reciprocal communication between PCCs [176].

#### 4.2.5. Macropinocytosis

Macropinocytosis is an endocytic process by which cancer cells can non-selectively uptake fluid and solid cargo for their nutrient sources. Macropinocytosis can be inhibited by an amiloride and its derivative, 5-(N-ethyl-N-isopropyl)-amiloride (EIPA). Amiloride can constrain macropinocytosis by impairing the activation of Rac1 and Cdc42 required for membrane ruffling [177]. In addition, treatments of EIPA showed the retardation of pancreatic cancer growth in vivo, presenting a potential strategy for pancreatic cancer therapy [178].

Furthermore, it was unveiled that CAFs can deliver valuable metabolites, such as amino acids, to cancer cells using exosomes under nutrient-deprived conditions. Exosomes are swallowed, at least in part, by macropinocytosis, and the growth of cancer cells invoked by these exosomes can be dampened using EIPA [5].

## 5. Conclusions

Accumulating evidence apparently demonstrated that a heterogeneous population of cells in cancer microenvironment shares their components via EV-mediated mutual communication, driving the malignancy of pancreatic cancer. Biologic constituents in EVs can be valuable targets for cancer therapy. It is still necessary to clarify the underlying mechanisms of cargo-mediated regulation of cellular signaling in pancreatic cancer to improve the therapeutic benefits of cargo targeting. For example, in the case of non-coding RNAs, it is indispensable to contemplate their features, such as dual roles and side effects [25]. In the case of miRNA-23b-3p, this miRNA facilitates the proliferation, migration and invasion of PCCs (Section 2.1.1.). However, another study demonstrated that there is a negative correlation between miRNA-23b-3p levels and radio-resistance in pancreatic cancer. MiRNA-23b-3p inhibits cytoprotective autophagy via targeting ATG12, sensitizing PCCs to radiation therapy [179]. These results indicate that miRNA-23b-3p regulates diverse cellular signaling pathways leading to different outcomes (stimulation of aggressive behaviors of PCCs vs radio-sensitization). In addition, it can be expected that the knockdown of miRNA-155 can reverse gemcitabine resistance and fibroblast phenotypes (Section 2.1.2. and Section 3.2.1.). However, inflammatory cytokines can be induced by miR-155 silencing in dendritic cells [180], suggesting the need to reckon specific cargo targeting in cancer to reduce conceivable side effects.

The development of strategies for the combination of cargo inhibitions with other anticancer agents can be another way to augment therapeutic responses. It is necessary to properly select candidate cargo molecules for positive treatment outcomes to achieve this goal. For example, is miRNA-146a a good candidate for inhibition? Although this miRNA is existing in CAF-derived exosomes that promote gemcitabine resistance [75], miRNA-146a is recently identified to hinder the proliferation and improve the efficacy of gemcitabine in PCCs [181].

Homeostasis of normal cells is also adjusted by EV-mediated exchange of cargo molecules. For example, harmful or excess molecules inside cells are secreted by EVs, contributing to preserving cellular homeostasis [182]. Therefore, the establishment of cancer targeted therapy will be required to less compromise normal cell circumstances.

Besides the EV-mediated dialogs, EVs are known to prompt drug export and neutralize the action of antibody-based therapeutic drugs [183,184], suggesting that interference with EV-mediated intercellular communication can be a promising strategy for pancreatic cancer treatments. Indeed, several studies have suggested the inhibitors controlling the biogenesis, release—or uptake—of EVs [10,185]. For the development of this therapeutic strategy, it will be required to evaluate the overall anticancer efficiency of EVs inhibition since it appears probable that blocking of exosome release can promote autophagy [186], which is generally cytoprotective in pancreatic cancer. In addition, the blocking of macropinocytosis and dynamin-dependent Endocytosis can perturb normal immune cell functions, such as antigen presentation. Advanced knowledge of the characteristics of EVs and their cargo molecules through future works will further provide a fundamental strategy for clinically valuable therapeutics.
